# Identifying the Heterogeneity in the Association between Workforce Diversity and Retention in Opioid Treatment among Black clients

**DOI:** 10.21203/rs.3.rs-3932153/v1

**Published:** 2024-02-08

**Authors:** Yinfei Kong, Erick Guerrero, Jemima Frimpong, Tenie Khachikian, Suojin Wang, Thomas D’Aunno, Daniel Howard

**Affiliations:** California State University; I-Lead Institute; New York University Abu Dhabi; Texas A&M University; Texas A&M University; New York University; Texas A&M University

**Keywords:** Workforce Diversity, Opioid Use Disorder, Treatment Retention, Causal Forest, Heterogeneous Treatment Effect

## Abstract

**Background:**

This study investigates the impact of workforce diversity, specifically staff identified as Black/African American, on retention in opioid use disorder (OUD) treatment, aiming to enhance patient outcomes. Employing a novel machine learning technique known as ‘causal forest,’ we explore heterogeneous treatment effects on retention.

**Methods:**

We relied on four waves of the National Drug Abuse Treatment System Survey (NDATSS), a nationally representative longitudinal dataset of treatment programs. We analyzed OUD program data from the years 2000, 2005, 2014 and 2017 (n = 627). Employing the ‘causal forest’ method, we analyzed the heterogeneity in the relationship between workforce diversity and retention in OUD treatment. Interviews with program directors and clinical supervisors provided the data for this study.

**Results:**

The results reveal diversity-related variations in the association with retention across 61 out of 627 OUD treatment programs (less than 10%). These programs, associated with positive impacts of workforce diversity, were more likely private-for-profit, newer, had lower percentages of Black and Latino clients, lower staff-to-client ratios, higher proportions of staff with graduate degrees, and lower percentages of unemployed clients.

**Conclusions:**

While workforce diversity is crucial, our findings underscore that it alone is insufficient for improving retention in addiction health services research. Programs with characteristics typically linked to positive outcomes are better positioned to maximize the benefits of a diverse workforce in client retention. This research has implications for policy and program design, guiding decisions on resource allocation and workforce diversity to enhance retention rates among Black clients with OUDs.

## Background

The opioid epidemic continues to take a significant toll on the public health system of the United States. The Centers for Disease Control and Prevention estimates that there were over 80,000 opioid-related overdose deaths in 2021.^1^ Increased OUD treatment retention can improve treatment outcomes, including reduced rates of mortality and of relapse.^2–5^ At the same time, retention rates in OUD treatment are highly variable between programs and demographic groups, with 6-month retention rates commonly dropping below 50%.^4,6^ Several studies measuring retention in OUD programs have found lower retention rates among individuals who identify as Black/African American and as Latino/Hispanic (Black and Latinos, hereafter).^7–9^ Other studies have identified subgroup differences between Black, Latino, and White clients, including variations in predictors of retention and the treatment outcomes associated with retention.^10–11^ It is therefore important to consider differences unique to Black clients when exploring strategies to boost retention rates in OUD programs.

Past research on the effect of culturally-responsive practices on the retention of Black OUD clients have identified promising culturally-responsive organizational factors, including offering: bilingual language services; developing specific policies and procedures designed to serve minority clients; and having managers who believe in the importance of cultural sensitivity.^12–16^ Workforce diversity, defined as having a higher percentages of Black staff members, is thought to improve Black OUD clients’ treatment outcomes by fostering a culturally-responsive treatment environment.^13,17–20^ However, previous studies on the impacts of workforce diversity on OUD client retention have looked for simple associations and have included only a few basic modifying variables, leading to variable retention outcomes.^16,21^

The heterogeneous nature of these results indicate that workforce diversity may have differential impacts on retention rates in OUD programs with different organizational characteristics. We build on prior studies that have suggested that workforce diversity in the absence of other factors, such as high levels of training and education among staff members, may be insufficient to improve treatment outcomes.^17,18^ Unpacking the heterogeneity in associations between workforce diversity and treatment retention can help healthcare policymakers, leaders of OUD treatment programs, and researchers to understand which programs would benefit most from the expansion of workforce diversity, and importantly, the additional conditions necessary to optimize the benefits of workforce diversity.

We apply heterogeneous treatment effect (HTE) estimation methods to understand which workforce diversity characteristics facilitate positive retention effects. HTE estimation is a machine learning method which was originally designed to study variations in the effects of clinical interventions, and has been generalized to other applications such as public policy, marketing, etc.^22–25^ In this paper, we adopted a state-of-the-art HTE estimation method called ‘causal forest,’ to examine the heterogeneous impact of workforce diversity on OUD treatment retention.^26,27^ There are several advantages of this method over traditional regression models. First, due to possible high collinearity and high false-discovery rate, we can only examine a limited number of interactions in traditional regression models. Second, causal forest provides variance for individually-estimated treatment effects, i.e., one can calculate the asymptotic *p* values for the statistical significance of treatment effects for each observation.

By examining HTE, we can untangle the various factors that may influence how workforce diversity impacts OUD client retention. The benefit of this study to the field of healthcare, and disparities within this field in particular, is to inform healthcare policy on which program characteristics can be adjusted to maximize the benefits of workforce diversity for OUD client retention. This study is also of relevance to the field of computational science, by using machine learning to showcase an application of a novel approach to understanding heterogeneity.

## Methods

We relied on nationally representative data from the National Drug Abuse Treatment System Survey (NDATSS), a dataset containing eight waves of survey data from outpatient substance use treatment programs (OTPs) from 1988–2017.^28,29^ Each wave incorporated a large percentage of programs from the previous wave, except programs excluded due to closure. More details on the NDATSS dataset can be found elsewhere.^21^ In this paper, we looked at the last four waves of the NDATSS (110 OTPs in 2000, 142 in 2005, 184 in 2014 and 190 in 2017).

### Dependent variables.

We used an established measure of retention, the percent of clients in treatment for more than three months. This measure has been used in other studies.^4,21,30^

### Independent variables.

The key independent variable is workforce diversity, which we define as percent of staff self-identified as Black or African American. This measure has been used in other studies as well. ^17, 18, 21,30^ To apply the existing estimation method for HTE, we dichotomized the treatment variable. Thus, we consider programs with more than 20% Black staff as having high workforce diversity. This threshold was chosen because more than 50% of the programs in our sample had less than 20% Black staff. The other independent variables that define the heterogeneity of the treatment effect on client retention rates include program and client characteristics such as: percent of Black clients, percent of Latino clients, accreditation by The Joint Commission (TJC), ownership status, program type (private-for-profit, private-not-for-profit, public), staff-to-client ratio, proportion of staff who have graduate degrees, percent of unemployed clients, and whether the program is located in a state that expanded Medicaid coverage.

### Statistical Analysis.

We conducted a comparative analysis of all variables across the four years using Chi-square tests or Analysis of Variance (ANOVA). To examine the heterogeneity of the association between workforce diversity and retention in OUD treatment, we used the causal forest method in which weights were incorporated to make the data nationally representative.^26,27^ The causal forest method can estimate the treatment effect that workforce diversity would have on retention for a given program. Causal forest also provides variance estimates to show if the treatment effect was significantly different from zero.

## Results

We found significant differences among variables across the four years examined. Table 1 presents the comparative analysis by year. The percentages of clients in treatment for more than 3 months were significantly different across years (p < 0.001). The percentages of Black clients were also significantly different across years (p < 0.001). More specifically, the percentages of Black clients were lower in the last two waves (2014 and 2017). More programs were from states that expanded Medicaid coverage in 2017 compared with 2014 (p < 0.001). There was an increasing trend of program age across years (p < 0.001). The results also showed that fewer programs were owned by another organization in the last two waves (p < 0.001). The staff-to-client ratio was significantly different across years (p = 0.024). The percentages of unemployed clients were higher in the last two waves (p < 0.001).

**Figure 1:**
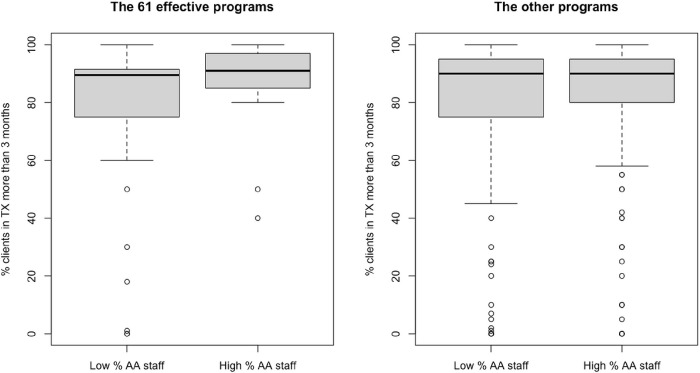
Left: box plots of percent of clients in treatment more than 3 months in high and low percent of Black staff in the 61 effective programs; Right: box plots of percent of clients in treatment more than 3 months in high and low percent of Black staff in the other 565 ineffective programs

Results from the causal forest method (Table 2) showed that sixty-one OTPs had statistically significant positive treatment effects. This means that these 61 OTPs would significantly benefit from having a high percent of Black staff in terms of increasing the percent of clients who stay in treatment longer than 3 months (retention). Among the remaining 566 OTPs, 562 did not have statistically significant treatment effects, while four had statistically significantly negative treatment effects. The comparison of characteristics of these 61 OTPs with the other 566 OTPs is presented in Table 2. The 61 OTPs that would benefit the most from workforce diversity had significantly lower percentages of Black clients (p < 0.001,), were more likely to be private-for-profit (p < 0.001), had lower staff-to-client ratio (p < 0.001), much higher proportion of staff who had graduate degrees (p < 0.001), much lower percentage of unemployed clients (p < 0.001), and were more likely to be newer programs (p < 0.001). The box plots of the percent of clients in treatment for more than 3 months by high and low percent of Black staff for these 61 OTPs and the other 566 OTPs are presented in Fig. 1. Higher percentages of Black staff increased the percent of clients in treatment to more than 3 months in these 61 OTPs.

## Discussion

To study the role of the variation of workforce diversity in improving OUD treatment retention, we explored the heterogeneous treatment effect with a novel machine learning method called causal forest. Our analytical method helped advanced understanding of the variation in the association between workforce diversity, i.e., percent of Black staff in an OUD treatment program, and OUD treatment retention (percent of clients in treatment for more than three months).

We found that only a small proportion of the sample, i.e., 61 out of 627 OTPs (less than 10%), would statistically significantly benefit from workforce diversity in retaining clients. It is important to note that the workforce of these 61 OTPs was not necessarily more diverse. Yet, their characteristics other than workforce diversity would cause them to benefit more from diversity. The characteristics that amplified the impact of workforce diversity on retention included: lower percentages of Black clients, lower staff-to-client ratio, higher proportion of staff who had graduate degrees, and lower percent of unemployed clients. In addition, those OTPs were more likely to be private for-profit and newer.

The characteristics of these 61 OTPs indicate that workforce diversity is most likely to improve client retention when implemented in less constrained programs, i.e., those with attributes often reported in the existing literature to be associated with positive outcomes.^4,30^ This may explain why we did not see a significant association between percent of Black staff and percent of clients in treatment for more than 3 months when considering the full sample of 627 OTPs.

Few studies have examined the general association between Black workforce diversity and treatment retention among Black clients.^16,21^ These studies identified significant associations that may have been driven by a small subgroup or population. Additionally, organizational characteristics may alter the impact of workforce diversity in a different direction. Findings in this paper inform rigorous analytical approaches to understand relationships between individual and program features and client outcomes. The benefit of this approach is to help public health policymakers identify OTPs that might benefit from workforce diversity, or alternatively, OTPs with high workforce diversity that could benefit from greater resources. Our study is also aligned with the national call to diversify the workforce in addiction health services, and thus informs how and when diversity could most benefit client-centered outcomes. Overall, identifying the heterogeneity of the relationship between workforce diversity and client outcomes in opioid treatment is an important first step to approaches that are more likely to better inform public health policy.

Policymakers should recognize that while workforce diversity is important, it is not a standalone solution for improving client retention in OUD treatment programs. Policies solely focused on increasing diversity may not yield desired outcomes unless other factors are addressed. This study highlights program characteristics associated with a positive impact of workforce diversity on retention, such as private-for-profit and newer programs with lower percentages of Black and Latino clients, lower staff-to-client ratios, higher proportions of staff with graduate degrees, and lower percentages of unemployed clients. Policymakers should allocate resources relating to these attributes to enhance the benefits of a diverse workforce. It is crucial to strike a balance between resource allocation and diversity goals, as less constrained programs, often linked to positive outcomes, maximize the benefits of diversity. Policies should support adequate resource allocation, including staffing and educational opportunities, while fostering diversity. Additionally, targeted strategies should prioritize retention rates among Black clients, addressing their unique challenges through tailored interventions, culturally competent care, and efforts to reduce disparities in access and quality of treatment. Overall, policies should consider program characteristics, resource allocation, and diversity goals to improve retention rates, particularly among Black clients, in OUD treatment programs.

## Limitations

Most existing methods can only estimate the heterogeneous treatment effects for binary variables. Thus, we had to dichotomize percent of Black staff to obtain a binary treatment variable. We chose the cutoff of 20% because 48.2% (i.e., about one half) of programs had more than 20% Black staff. Ideally, we would explore the heterogeneity with the original continuous variable, i.e., percent of Black staff. The identified 61 OTPs would have a greater impact on retention given their diverse workforce. However, there may be other heterogeneity among these 61 OTPs in the association between workforce diversity and retention. We did not examine such heterogeneity in this paper because the higher treatment effects on these 61 OTPs were composite effects of several variables. In fact, we cannot observe significant treatment effects by altering the value of just one variable, while keeping the others constant. Moreover, our finding that lower percentage of Black clients being associated with lower constraints, and therefore greater retention, should be further examined. Future studies should scrutinize this finding to better understand the mechanisms that drive this association, and approaches to improve outcomes equally and equitably.

## Conclusions

Our findings expand our understanding of the role that workforce diversity, in the form of higher percentages of Black staff, plays in enhancing retention in opioid treatment among Black clients. It is critical to rely on advanced statistical methods to account for and address when diversity benefits clients, and especially minority clients, vis-a-vis program resources to serve minority communities. As federal and state authorities prepare to deliver a significant influx of financial resources drawn from pharmaceutical settlements and new taxation revenues to enhance access to opioid treatment,^31,32^ it is critical to know how to best support OTPs improve patient outcomes in general, and among minority populations in particular.

## Figures and Tables

**Table 1.** Comparative analysis of Opioid Treatment Programs in NDATSS data

	2000 (N = 110)	2005(N = 142)	2014 (N = 184)	2017 (N = 190)
Percent of clients in treatment more than 3 months***	84.3 (16.5)	87.9 (13.2)	75.2 (30.5)	79.0 (27.7)
More than 20% Black staff	61 (55.5%)	70 (49.3%)	78 (42.4%)	93 (48.9%)
Client characteristics				
Percent of Black clients***	29.4 (28.1)	25.5 (26.4)	18.7 (24.1)	20.8 (23.0)
Program characteristics				
Medicaid expansion***	--	--	116 (63%)	140 (73.7%)
TJC accreditation	27 (24.5%)	52 (36.6%)	60 (32.6%)	55 (28.9%)
Program age***	17.8 (11.6)	20.8 (12.0)	23.7 (14.1)	27.0 (15.1)
Owned by another organization***	78 (70.9%)	103 (72.5%)	43 (23.4%)	59 (31.1 %)
Type of programs				
Private for-profit	40 (36.4%)	53 (37.3%)	63 (34.2%)	64 (33.7%)
Private not-for-profit	45 (40.9%)	64 (45.1%)	101 (54.9%)	99 (52.1 %)
Public	25 (22.7%)	25 (17.6%)	20 (10.9%)	27 (14.2%)
Staff-to-client ratio in percentage*	4.3 (4.1)	3.9 (2.7)	4.2 (4.4)	6.1 (10.6)
Proportion of graduate staff	0.3 (0.2)	0.4 (0.2)	0.3 (0.2)	0.3 (0.2)
Percent of unemployed clients***	44.6 (23.0)	43.6 (24.7)	54.7 (26.4)	52.1 (25.8)

* p < 0.05

** p < 0.01

*** p < 0.001

**Table 2.** Comparative analysis of programs with no or significant benefit from workforce diversity

	No Benefit from Diversity (N = 565)	Benefit from Diversity (N = 61)	p value
Medicaid Expansion	233 (41.2%)	23 (37.7%)	0.69188
Year			0.34624
2000	104 (18.4%)	6 (9.8%)	
2005	129 (22.8%)	13 (21.3%)	
2014	163 (28.8%)	21 (34.4%)	
2017	169 (29.9%)	21 (34.4%)	
Percent of Black clients	24.9 (25.7)	2.6 (2.9)	2.12E-66
TJC accreditation	182 (32.2%)	12 (19.7%)	0.06199
Owned by another organization	261 (46.2%)	22 (36.1%)	0.16922
Type of programs			2.22E-10
Private for-profit	175 (31%)	45 (73.8%)	
Private not-for-profit	298 (52.7%)	11 (18%)	
Public	92 (16.3%)	5 (8.2%)	
Staff-to-client ratio in percentage	5.0 (7.0)	2.0 (1.1)	2.38E-19
Proportion of graduate staff	0.3 (0.2)	0.5 (0.2)	2.43E-05
Percent of unemployed clients	52.0 (25.2)	27.1 (17.8)	4.31 E-16
Program age	23.9 (13.9)	14.5 (10.8)	1.51E-08

## Data Availability

The datasets, the National Drug Abuse Treatment System Survey (NDATSS), used and/or analyzed during the current study are available from the co-author Thomas D’Aunno.

## What is known on this topic

- Existing research suggests OUD treatment retention is associated with a variety of factors at both individual and program level.
- Clients may stay in OUD treatment for shorter or longer periods of time depending on what programs they receive treatment from.
- Analysis of the variation in the association between workforce diversity and OUD treatment retention is difficult when using traditional methods, such as, regression.

## What this study adds

- The machine learning method, ‘causal forest’, provides the estimate of association for each observation, identifying heterogeneous association between workforce diversity and retention in OUD treatment more accurately than linear regression methods.
- This study identifies the characteristics of OUD treatment programs that can statistically significantly benefit from the workforce diversity.
- Programs that had a positive impact of workforce diversity on client retention included those that were more likely private-or-profit, newer, and had lower percentages of Black clients, lower staff-to-client ratios, higher portions of staff who have graduate degrees, and lower percentages of unemployed clients.

## List Of Abbreviations

OUD: Opioid use disorder
NDATSS: National Drug Abuse Treatment System Survey
HTE: heterogeneous treatment effect
OTPs: outpatient substance use treatment programs
TJC: The Joint Commission
ANOVA: Analysis of Variance
